# Distinct Mechanisms of Inadequate Erythropoiesis Induced by Tumor Necrosis Factor Alpha or Malarial Pigment

**DOI:** 10.1371/journal.pone.0119836

**Published:** 2015-03-17

**Authors:** Abigail A. Lamikanra, Alison T. Merryweather-Clarke, Alex J. Tipping, David J. Roberts

**Affiliations:** 1 Nuffield Division of Clinical Laboratory Sciences, University of Oxford, Oxford OX3 9BQ, United Kingdom; 2 National Health Service Blood and Transplant, John Radcliffe Hospital, Headington, Oxford OX3 9BQ, United Kingdom; Southern Illinois University School of Medicine, UNITED STATES

## Abstract

The role of infection in erythropoietic dysfunction is poorly understood. In children with *P*. *falciparum* malaria, the by-product of hemoglobin digestion in infected red cells (hemozoin) is associated with the severity of anemia which is independent of circulating levels of the inflammatory cytokine tumor necrosis alpha (TNF-α). To gain insight into the common and specific effects of TNF-α and hemozoin on erythropoiesis, we studied the gene expression profile of purified primary erythroid cultures exposed to either TNF-α (10ng/ml) or to hemozoin (12.5μg/ml heme units) for 24 hours. Perturbed gene function was assessed using co-annotation of associated gene ontologies and expression of selected genes representative of the profile observed was confirmed by real time PCR (rtPCR). The changes in gene expression induced by each agent were largely distinct; many of the genes significantly modulated by TNF-α were not affected by hemozoin. The genes modulated by TNF-α were significantly enriched for those encoding proteins involved in the control of type 1 interferon signalling and the immune response to viral infection. In contrast, genes induced by hemozoin were significantly enriched for functional roles in regulation of transcription and apoptosis. Further analyses by rtPCR revealed that hemozoin increases expression of transcription factors that form part of the integrated stress response which is accompanied by reduced expression of genes involved in DNA repair. This study confirms that hemozoin induces cellular stress on erythroblasts that is additional to and distinct from responses to inflammatory cytokines and identifies new genes that may be involved in the pathogenesis of severe malarial anemia. More generally the respective transcription profiles highlight the varied mechanisms through which erythropoiesis may be disrupted during infectious disease.

## Introduction

Although recent efforts to reduce the number of deaths due to malaria have had some success, there is still an estimated 1.2 billion people at high risk of infection worldwide with almost half a million deaths occurring in children. The majority of these infections are due to *P*. *falciparum* and *P*. *vivax* [1]. Most mortality is caused by *P*. *falciparum* or mixed infections of *Plasmodia* including falciparum where the majority of hospital admissions in endemic regions are of children under the age of four [2, 3].

In young infants in holo-endemic regions of Africa, the predominant syndrome of severe malaria is severe malarial anemia (reviewed in [4, 5]). The recently observed elevated levels of hepcidin in patients with acute *P*. *falciparum* malaria suggest that the reduced bioavailability of iron contributes to developing severe anemia [6, 7]. Severe malarial anemia is due not only to increased hemolysis of infected and non-infected red blood cells but also to a striking degree of abnormal development of erythroid precursors in acute and chronic infection [8, 9] and an inadequate erythropoietic response in spite of elevated levels of erythropoietin (Epo) [9–11]. The distribution of erythroid precursors in the cell-cycle is also abnormal with an increased number of cells in the G_2_ phase compared with normal controls [12, 13].

Severe malaria is characterized by elevated levels of the inflammatory cytokine TNF-α [14, 15] which is thought to be produced following phagocytosis of malarial pigment (hemozoin) by macrophages [16]. Hemozoin is formed in the food vacuole of developing intra-erythrocytic parasites, as toxic heme remaining after digestion of hemoglobin forms a crystalline dimer of α hematin, complexed with lipid and protein. Hemozoin crystals closely resemble β hematin, consisting of a ferric ion within a protoporphyrin IX ring structure [17]. Hemozoin released after the lysis of infected red blood cells is heterogeneous and associated with proteins, nucleic acids, and host- and parasite- derived lipids including products from lipid peroxidation such as 4-hydroxy-2-nonenal (HNE) [18, 19].

Although the link between TNF-α and bone marrow suppression in anemia of chronic disease such as rheumatoid arthritis is well documented [20], the inadequate response of the bone marrow during severe malarial anemia can be attributed to factors other than TNF-α. In clinical studies of children with malarial anemia, the proportion of circulating monocytes containing hemozoin and levels of plasma hemozoin were associated with anemia and reticulocyte suppression, independently of the level of circulating cytokines, including TNF-α. Furthermore, histologic examination of the bone marrow of children who have died from malaria shows that pigmented erythroid and myeloid precursors are associated with the degree of abnormal erythroid development [9]. Taken together, these observations provide compelling evidence for independent inhibition of erythropoiesis by hemozoin and TNF-α.

Extensive examination of clinical bone marrow samples is difficult for ethical reasons but *in vitro* studies have shown that suppression of erythroid precursor expansion by TNF-α is mediated by induction of nuclear factor kappa B (NF-κB) that inhibits erythroid-specific gene expression [21] and reviewed in [22]. Addition of extracted hemozoin to TNF-α enhances inhibition by TNF-α alone [9]. However hemozoin can induce apoptosis in the absence of inflammatory cytokines [23] and HNE can cause cell cycle arrest in erythroid progenitors [24].

These observations suggest that hemozoin and TNF-α exert their inhibitory effects on erythroblasts via different molecular pathways, but may act additively on developing erythroid cells. To better understand the pathology of severe malarial anemia we wanted to determine if there are molecular signatures for dyserythropoiesis and/or inadequate erythropoiesis that can distinguish between the effects of hemozoin and TNF-α, and to understand if their inhibitory effects in terms of modulating gene expression are overlapping or largely distinct. To do this, we used gene expression profiling by microarray to compare the transcriptome of purified erythroblasts incubated with either TNF-α or hemozoin extracted from *P*. *falciparum* cultures.

## Results

### Development of erythroid cultures exposed to hemozoin or TNF-α

**Fig 1:**
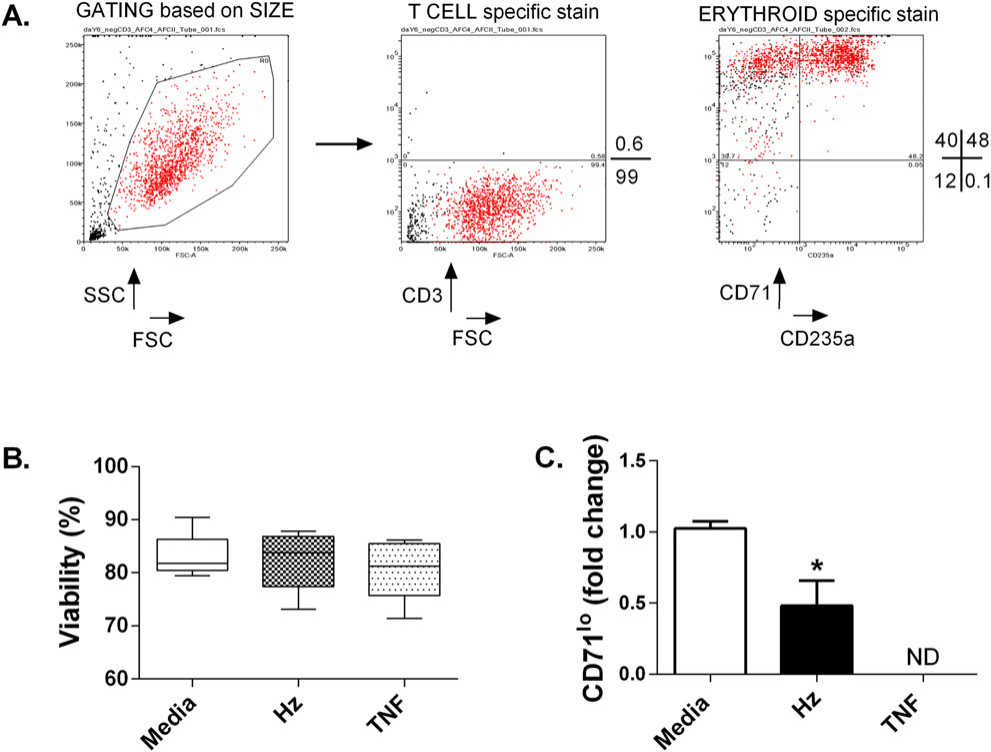
Representative example of samples used for microarray and rtPCR. The purity of erythroblast populations treated with either TNF-α or hemozoin was determined by flow cytometry (A). The forward scatter (FSC) and side scatter (SSC) of live cells is shown in red. The proportion of cells (% of total events acquired) expressing lymphoid (CD3) and erythroid markers (CD71^-^CD235a^+^ and CD71^+^CD235a^+^) is represented by values to the right of each plot. The average response to treatment of all samples after 24 hours in which viability was assessed (B) or after 6 days when maturation was assessed by comparing the proportion of committed erythroid progenitors (CD235a^+^) that express low levels of CD71 (CD71^lo^) (C). Error bars show standard error of the mean of responses from 4 different donors; ND, not determined due to insufficient cell recovery. *p≤0.05 when compared with media alone (two tailed student’s test).

The viable erythroblasts used for microarray comprised 97% early pro-erythroblasts (CD71^high^CD235a^-^) and the more mature intermediate stage of erythroid development (CD71^high^CD235a^+^) with negligible contamination from other lineages (Fig. 1a and S1 Dataset). These erythroblasts were isolated from cultures derived from different donors and incubated with either 12.5 μg/ml heme equivalents of hemozoin or 10ng/ml TNF-α, doses previously shown to inhibit erythroblasts derived from adult peripheral blood [9, 23]. The viability of erythroid cells derived from different donor cultures used for microarray and qPCR were unchanged at 24 hours (Fig. 1b), but after 6 days the proportion of erythroid progenitors (CD235a^+^) that had matured and were expressing low levels of CD71 (CD71^lo^) (Fig. 1c) was reduced by 40% in cultures treated with hemozoin. This supports previous observations that this dose and preparation of hemozoin can inhibit erythroid expansion and development and so warranted further investigation to determine if the mechanism(s) that underlie its effect differ from those induced by TNF-α.

### Gene expression profiles in erythroid cultures exposed to hemozoin or TNF-α

Erythroblasts incubated with either 12.5 μg/ml heme equivalents of hemozoin or 10ng/ml TNF-α were harvested after 24 hours and total RNA extracted. Following confirmation of inhibition by hemozoin or TNF-α after 6 days. Biotinylated fragmented cRNA was synthesized and hybridized to Affymetrix GeneChip Human Genome arrays (HG-U133_plus _2.0).

Data was normalized using RMA [25] and filtered to select probes with a signal of at least 100 in any one sample. To examine changes in response to hemozoin and TNF-α exposure, the dataset was filtered for genes changing greater than 1.5-fold in response to hemozoin and was hierarchically clustered by sample and by gene together with data previously acquired from erythroblasts sorted and grouped by stage of erythroid development using cell surface markers (CD36, CD71 and CD235a) and morphology [26]. The list of differentially expressed genes is given in Supporting Information 1 (S1 Dataset).

**Fig 2:**
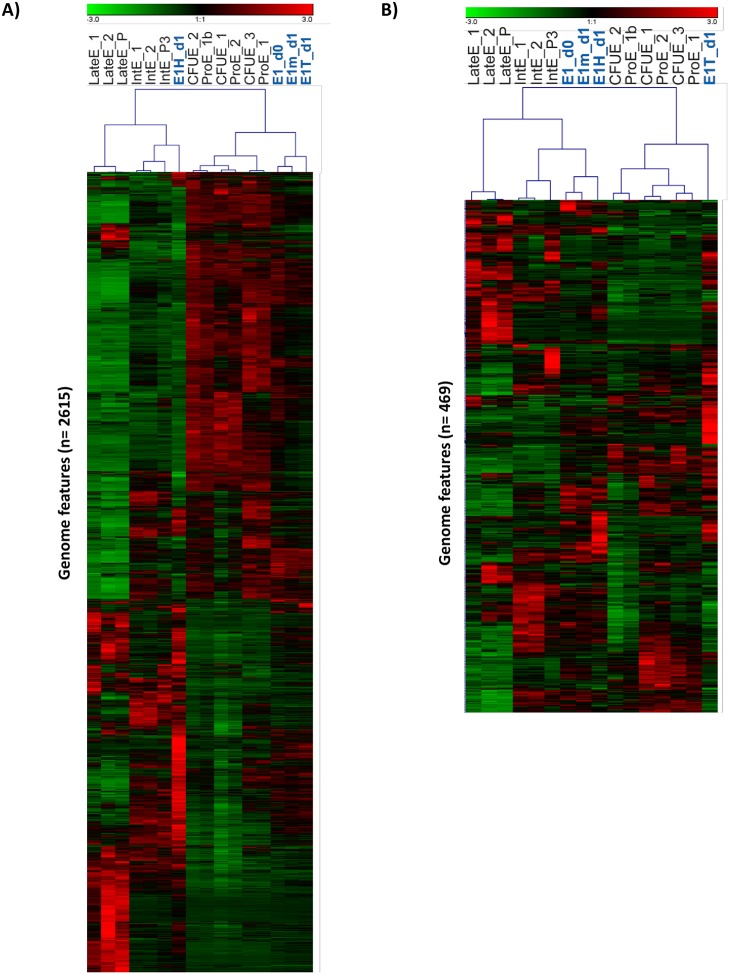
Hierarchical clustering. Expression of genes modulated >1.5x by hemozoin in erythroid cells before treatment (E1_d0), after incubation for 24 hours with hemozoin (E1H_d1) or TNF-α (E1T_d1) and without treatment in culture medium (E1m_d1) are shown, together with previously described sorted populations of erythroblasts [26]. Hierarchical clustering by gene and experiment (Pearson correlation) is shown centred around these hemozoin- modulated genes (A) or TNF-α- modulated genes (B). CFUE, colony forming unit erythroid FACsorted; ProE, pro- erythroblast FACsorted; Int, Intermediate erythroblast FACsorted; LateE, Late erythroblast sorted; E1, magnetic bead sorted erythroblasts; m, medium; T, TNF-α; H, hemozoin.

When considering all genes irrespective of differential regulation, treated and untreated samples clustered together, closest to the intermediate stage of primary samples (S1 Fig.). However when considering only those genes regulated by hemozoin, the separation of the gene expression profiles of hemozoin- and TNF-treated erythroblasts in the first bifurcation of the sample clustering dendrogram suggested that the gene expression program unfolding in response to hemozoin is quite different from that of untreated and TNF-treated counterparts (Fig. 2a). Similarly, repeating this analysis centered on the genes modulated by TNF-α treatment clustered the TNF-treated cells away from their untreated counterparts (Fig. 2b). Notably, in both cases genes differentially regulated by the agent under study are not similarly affected by the other agent; i.e. cells treated with hemozoin cluster together with untreated cells when examining the TNF-α differential genes, and vice versa.

### Functional associations between differentially regulated genes and biological processes

We first examined differential gene expression within each treatment group by comparing genes that were up- or down-regulated by at least 1.5 fold in response to each treatment. The statistical significance of functional associations between genes and biological processes within treatment groups were then determined using Genecodis-3 Hypergeometric analyses, corrected using a false determination rate calculation [27].

### Response of erythroid cultures to TNF-α

**Fig 3:**
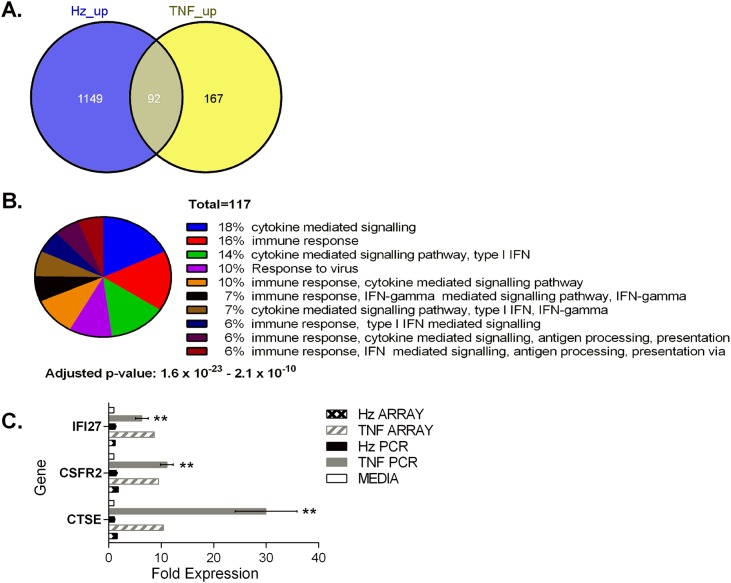
TNF-α induces up-regulation of genes distinct to those induced by hemozoin. Venn analysis illustrates the overlap in genes up-regulated following treatment of primary erythroid cultures. Primary cultures of erythroblasts were established from peripheral blood and basophilic (CD71^+^CD235a^-^ and CD71^+^CD235a^+^) erythroblasts isolated as described previously [23]. Erythroblasts were incubated with 10ng/ml TNF-α or 12.5μg/ml hematin units of hemozoin for 24 hours (A). Pie chart showing the number of up-regulated genes associated with biological processes described per concurrent annotation following treatment with TNF-α. The number of genes with annotations that fall within the hypothetical p values shown are listed, together with the proportion that they represent (B). Confirmation of distinct patterns of up-regulation by TNF-α observed in microarray by rtPCR using TaqMan gene expression assays (C). Fold expression compared with cells cultured in media alone is shown. TNF, TNF-α; Hz, hemozoin. Statistical analysis of rtPCR data was performed using the two tailed student’s t test. ** p<0.05 when compared with media or hemozoin. Fold expression determined from the original array data is shown for comparison.

The most striking finding was that the majority of genes up-regulated by TNF-α were not differentially expressed in erythroblasts incubated with hemozoin (Fig. 3a and Table A in S1 Dataset), consistent with the clustering analysis. Of the 259 genes up-regulated by TNF-α approximately 64% of these were not significantly induced by hemozoin. As expected, gene ontology analysis revealed that more than 80% of the genes induced in erythroblasts treated with TNF-α are involved in innate and adaptive immune responses to infection (p<10^-15^) (Fig. 3b and Table B in S1 Dataset). Only 8% of the 1241 genes up-regulated by hemozoin were induced in erythroblasts treated with TNF-α. Amongst the up-regulated genes associated with immune function are Cathepsin E *(CTSE)*, colony stimulating factor receptor 2 (*CSF2RB*) and interferon α-inducible protein 27 (*IFI27*). *CTSE* and *IFI27* are involved in proteolysis of antigens and cytokine mediated signaling respectively. *CSF2RB* is the signaling sub-unit (βc) for the IL-3, IL-5 and GMCSF family of receptors reviewed in [28] and is required for cell growth, adaptive immune responses and myeloid differentiation. However further analysis by qPCR using erythroblasts from additional donors showed that up-regulation of these TNF-α up genes by hemozoin was insignificant when compared with the up-regulation induced by the pro-inflammatory cytokine TNF-α (Fig. 3c).

### Response of erythroid cultures to hemozoin

**Fig 4:**
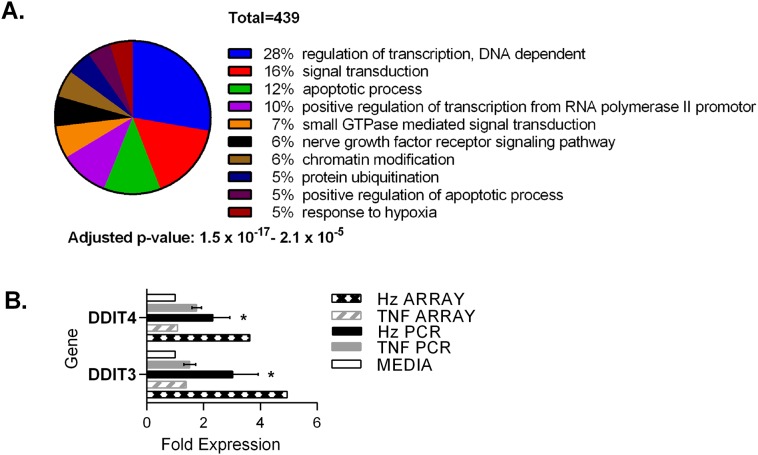
Up-regulated genes induced by hemozoin. Pie chart showing the number of up-regulated genes associated with biological processes following treatment with hemozoin (A). Confirmation of observations in microarray data by rtPCR using TaqMan gene expression assays (B). Fold expression compared with cells cultured in media alone is shown. TNF, TNF-α; Hz, hemozoin. Statistical analysis of rtPCR data was performed using the two tailed student’s t test. * p≤0.05 when compared with media alone. Fold expression determined from the original array data is shown for comparison.

Statistical analyses showed that the majority of genes up-regulated by hemozoin are required for control of gene expression (p = 10^-17^) e.g. transcription factors controlling red cell differentiation and apoptosis whilst others were enriched in processes required for survival during cellular stress, neurotrophin tyrosine receptor kinase signaling and protein ubiquitination (p<10^-7^) (Fig. 4a and Table D in S1 Dataset). Genes up-regulated by TNF-α were not significantly associated with these gene ontology categories (Table B in S1 Dataset).

### Inhibition of erythropoiesis by hemozoin

We wanted to identify differential expression that could explain inhibition of erythropoiesis by hemozoin. An example of up-regulation that could alter erythroid gene expression and differentiation is *DDIT3*, the C/EBP homologous protein (CHOP), a transcription factor that also mediates growth arrest and apoptosis as part of the integrated stress response to misfolded proteins in the ER (reviewed in [29]). Of note, also induced by hemozoin was the related gene *DDIT4* (DNA damage-induced transcript 4 (*REDD1*) which can be induced by p53 in response to DNA damage and also forms part of the integrated stress response [30]. Further rtPCR examination of induced expression in erythroblasts from four donors confirmed significant induction of *DDIT3* and *DDIT4* transcripts by hemozoin which was absent in cultures incubated with TNF-α (Fig. 4b).

### Down-regulation of genes in reponse to TNF-α or hemozoin

**Fig 5:**
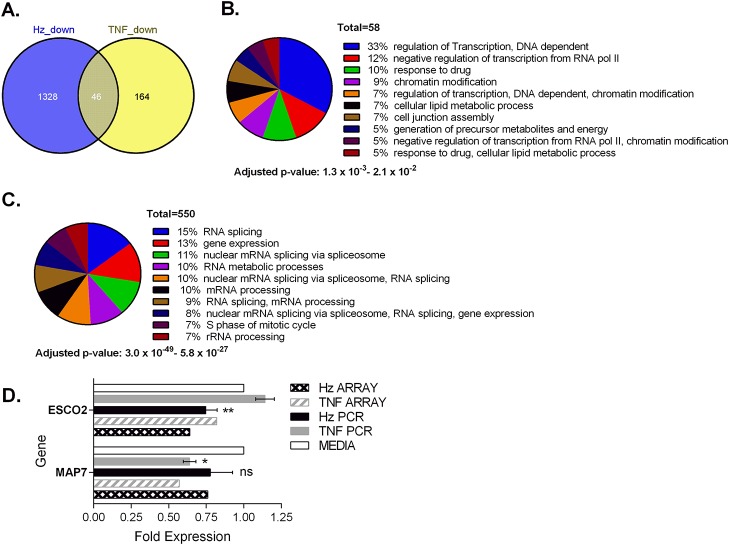
Hemozoin induces down-regulation of genes distinct to those affected by TNF-α. Venn analysis illustrates the overlap in genes down-regulated following treatment of erythroid cultures (A). Pie charts that show the number of down-regulated genes associated with biological processes described per concurrent annotation following treatment with TNF-α (B) or hemozoin (C). The number of genes with annotations that fall within the hypothetical p values shown are listed together with the proportion that they represent. Microarray observations were confirmed by rtPCR for microtubule associated protein 7 (MAP7) and establishment of cohesion 2 (ESCO2) (D). Statistical analysis of rtPCR data was performed using the two tailed student’s t test. ** p<0.05 when compared with media or TNF-α, * p≤ 0.05 when compared with media alone; ns, non-significant compared with media.

Of the 210 genes down-regulated by TNF-α, 78% were not significantly modulated by hemozoin and returned weak functional associations with relatively insignificant corrected p values above 10^-3^ (Figs. 5a, 5b and Table F in S1 Dataset). Conversely, statistical analysis of the 1328 genes down-regulated by hemozoin returned annotations that revealed a significant enrichment of genes known to encode for proteins involved in RNA splicing, ribosome biogenesis and the mitotic cell cycle. All of these predictions had corrected hypothetical probabilities of 10^-10^ or less (Fig. 5c and Table G in S1 Dataset). Genes down-modulated by both hemozoin and TNF-α were weakly associated with ontologies including cell cycle and nuclear mRNA splicing via the spliceosome with p values > 10^-3^ (Table J in S1 Dataset) possibly suggesting some degree of coalescence of these down-regulated programs at the functional level.

Regulation of the spliceosome apparatus is closely linked to the cell cycle [31] and since hemozoin and its by-products are known to induce cell cycle arrest, we were interested to know if treatment of cells with hemozoin or TNF-α affected genes known to be important during mitosis. The microarray data showed that a gene required for stabilisation of the cytoskeleton during mitosis, microtubule associated protein 7 (MAP7/ensconsin) [32, 33], was reduced almost 2 fold by TNF-α and reduced 1.5 fold by hemozoin. The expression of another gene active during mitosis ESCO2 (establishment of sister chromatid cohesion 2) was reduced 1.5 fold by hemozoin, but remained unaffected by TNF-α. Further confirmatory rtPCR on treated cultures derived from 4 donors confirmed that MAP7 was more markedly and significantly reduced by TNF-α (Fig. 5d). Conversely ESCO2 was significantly reduced by hemozoin (p = 0.029) but remained unchanged or increased with TNF-α.

## Discussion

The analysis of gene expression in erythroid cells described here provides, for the first time, a mechanistic insight into the different pathways that can lead to dyserythropoiesis and erythroid death during a malaria infection. This has been achieved by comparison of carefully temporally- and phenotypically-defined populations of erythroid cells exposed to TNF-α. Notably, hemozoin treatment that reduced viability and maturation of erythroblasts did not induce expression of TNF-α or ontologically-linked target genes in the transcriptional data, supporting the idea that although both agents can induce dyserythropoiesis, this is achieved via largely independent transcriptional programs.

There were at least seven times as many genes uniquely regulated by hemozoin as there were by TNF-α. However, in contrast to TNF-α few of the observed gene expression changes modulated by hemozoin were directly associated with immune function. Interestingly, changes in the transcriptome that indicate increased DNA damage and misfolding of proteins in the ER were prominent. This is consistent with previous observations that describe increased oxidative stress, apoptosis and/or cell cycle arrest in erythroid progenitors incubated with hemozoin or lipids associated with hemozoin, such as 4-hydroxy-nonenal (HNE) [23, 24].

The transcriptional profile following treatment of erythroblasts with either TNF-α or hemozoin shares some overlap with previous observations in peripheral blood of children with acute malaria [34] and in murine models of malaria [35]. In these studies the gene expression signature would be influenced by multiple cell types exposed to different components of the parasite’s life- cycle.

By using highly-enriched and purified erythroid cultures in which all viable responsive cells comprised hematopoietic erythroid precursors (Fig. 1a; S1 Tables), we were able to focus on the effects of hemozoin on erythroid gene expression without the confounding contribution from gene expression signatures of other contaminating cell lineages. Previously, a similar study has observed less potent inhibition of cell expansion by hemozoin but a greater effect on erythroid commitment when compared with TNF-α [36]. Although the erythroblast stage of development, culture conditions and hemozoin preparation used in this study differ from those described in the work presented here these observations are also consistent with the suggestion that distinct molecular pathways are affected by hemozoin and TNF-α.

In our study we have used physiologically relevant quantities of native hemozoin and TNF-α that were capable of inducing modest cell death. This aimed to mimic the scenario in the bone marrow where macrophages and neutrophils are unable to scavenge pigment due to inhibition by ingested trophozoites [19]. Indeed a recent study has shown that free hemozoin is present in the bone marrow which would support this hypothesis [37]. Since the majority of responsive cells in our cultures were erythroblasts, the role of macrophages would have been minimal (S1 Tables) such that the entry of hemozoin into erythroblasts would likely be via endocytic pits [38]. It is possible that with non-physiological higher doses, the effects of hemozoin on the erythroid transcriptome might differ from that described here, and might yield higher fold changes in gene expression but with the risk of inducing non-specific effects. It could be argued that pooling both early CD235a^-^ and more mature CD235a^+^ proliferating CD71^+^ progenitors provided a less distinct pattern of gene expression than would be seen if these populations were separated. A future approach to achieve this beyond the scope of this study would be to use a fluorescence activated sorter to separate these populations.

Notwithstanding these points, we were able to identify and confirm distinguishable gene expression program changes induced by each of the two toxins on erythroblasts that may discriminate between the pathophysiological features of anemia due to inflammation, and those due to *P*. *falciparum* infection.

Previous work shows that four days treatment with TNF-α inhibits erythroid development by changing the balance of expression of the cross-antagonistic lineage-affiliated transcription factors GATA-1 (erythroid) and PU.1 (myeloid), thereby reducing the expression of GATA-responsive genes such as those encoding the Epo receptor and the hemoglobin α and β chains [39]. Here, we did not observe these changes, which may be partly due to differences in the timing of the experiment, or possibly that the probes used on the array may not optimally detect these genes [40]. However we did observe reversal of the expected pattern of erythroid gene expression in TNF-treated erythroid cells, in that expression of *CSF2RB* was increased, where normally it is decreased during erythropoiesis. Moreover, the pro-apoptotic gene *IFI27* was induced by TNF-α when normally it should diminish during erythropoiesis [41]. These observations are consistent with activation of NF-_κ_B downstream of TNF-α binding to TNFR1 [42]. The induction of *CSF2RB* by TNF-α could potentially result in formation of the tissue protective form of the Epo receptor or the receptor for GMCSF that supports myeloid differentiation [28, 43, 44]. Any one of these changes could arrest development of the erythroid precursors used in this study and prevent their progression to mature stages.

During erythropoiesis *in vitro* expression of both *DDIT4* and *DDIT3* increases from CFU-e through to the intermediate and late stages of erythroid differentiation, and *ESCO2* drops significantly at the latest stage of development [41]. After 24 hours in culture with hemozoin, we found that mRNA transcripts for *DDIT4* and *DDIT3* were significantly increased and the transcripts for *ESCO2* were prematurely decreased, compared with media controls or cells treated with TNF-α. These changes would therefore be expected to contribute to the clustering of pro-erythroblasts towards more mature intermediate erythroblasts following incubation with hemozoin for 24 hours (see Fig. 2).

We have previously shown that hemozoin mediates mitochondrial depolarization and apoptosis in erythroblasts [23] which can be mediated by increases in ROS and DNA damage respectively. Our observations by microarray, confirmed by rtPCR, are consistent with this. DDIT4 can increase the cell’s sensitivity to ROS resulting in cell death [30]. During cellular stress, especially after misfolding of proteins in the ER, DDIT3 accumulates in the nucleus where it transactivates the proapoptotic activating protein 1 (AP-1) complex [45]. DDIT3 can also inhibit the induction of Bcl2 [46] which allows accumulation of Bax and Bak in mitochondrial membranes leading to mitochondrial permeability and apoptosis. ESCO2 is required for cohesion during replication and repair of double strand breaks caused by DNA damage [47]. Loss of function mutations in ESCO2 result in hypersensitivity to DNA damaging reagents and reduced cell expansion [48] which may be mediated by activation of caspase 8 following G_2_ arrest [49]. The observed down-regulation of ESCO2 transcripts shown here therefore agrees with the previously described cell cycle arrest and induction of cleaved caspase 8 by hemozoin [23, 24].

The expression of DDIT4 is consistent with up regulation of p53 activity. Indeed induced expression of *TP53INP1* (p53 induced nuclear protein 1) which mediates apoptosis [50] and reduced expression of *TP53BP1* (tumor protein p53 binding protein) required for the repair of double stranded breaks in DNA [51], was seen in erythroblasts treated with hemozoin (Tables C and G in S1 Dataset). Furthermore, we observed down-modulation of the minichromosome maintenance complex (MCM) family of genes [52] by hemozoin and not TNF-α supporting an increase in p53 activity due to DNA damage [53] (Table K in S1 Dataset).

Other investigators have shown that the highly reactive component of hemozoin, HNE, can also alter expression of genes required for cell cycle checkpoint signalling and repair of DNA damage in a macrophage cell line [54]. In contrast to the work described here, they also observed up-regulation of pro-inflammatory cytokines such as TNF-α in response to HNE and IL-1β in response to β-hematin. This difference could be attributed to one or all of the following: differential gene expression programs extant in macrophages and erythroid cells driving differing responses; intrinsic properties of the macrophage cell line used; the maturation step using lipopolysaccharide that also activates the innate immune response; or the dose of HNE used which was equivalent to 100μM native hemozoin. The work described here uses 10 fold less native hemozoin on primary cultures of erythroblasts and thus, we believe, would more closely reflect the responses of developing erythroid cells. We (S2 Fig.) and others [55] have observed induction of DDIT3 protein by HNE. These observations suggest that HNE may in part contribute to the induction of integrated stress response by hemozoin in erythroblasts and therefore warrants further investigation in the context of malarial anemia.

The gene expression profile of primary erythroblasts infected with the early ring stage of *P*. *falciparum* has also revealed transcriptional changes in the host associated with regulation of reactive oxygen species and ER stress [56]. Of particular note is that genes required for cleavage and polyadenylation of pre-mRNA were specifically up-regulated in response to *P*. *falciparum*. The authors suggest this could provide nutrient and metabolic factors to the parasite. As such the transcriptional changes described would therefore include host responses to the parasite developing within the erythroblast. In this study we used an isolated component from later stages of the parasite’s life cycle to exclude direct effects of the parasite on erythropoiesis.

Taken together, the gene expression described in these studies, suggest multiple factors that can contribute to ineffective erythropoiesis in severe malarial anemia: hemozoin-induced repression of macrophage function; secretion of pro-inflammatory cytokines from macrophages; and perturbation of erythroblast metabolism to reduce red cell output from the bone marrow.

By comparing the effects of the parasite product hemozoin with the inflammatory mediator TNF-α on primary erythroblasts, we were able to distinguish between some of the effects of the host’s immune response to infection and that of hemozoin on the erythroid transcriptome. We found that largely distinct molecular programs were induced by hemozoin from those induced by TNF-α. The response to hemozoin was associated more with defects in the DNA repair response and a greater activity within the ER stress response pathway similar to that seen in macrophages incubated with HNE [54].

## Conclusion

The work described here therefore provides further insight into the mechanisms that can mediate severe malarial anemia by identifying components of the integrated stress response pathway that can be investigated further to better understand the etiology of bone marrow suppression due to *P*. *falciparum* infection. Future studies will therefore target specific steps within the stress response and DNA repair pathway of erythroblasts and macrophages to reverse inhibition of erythropoiesis.

## Methods

All tissue culture grade reagents were obtained from (Sigma, Poole, UK) unless otherwise stated. Peripheral blood mononuclear cells (PBMCs) were obtained from blood donated to the National Health Service Blood and Transplant (NHSBT; www.nhsbt.nhs.uk) following written consent and approval of their use by the National Health Service Oxfordshire Regional Ethical Committee.

### Tissue Culture

The 3D7 strain of *P*. *falciparum* was up to 10% parasitemia at 1–2% hematocrit as described previously [57, 58]. Erythroid cultures were grown from PBMCs in a two-stage system [59]. Briefly, PBMCs were isolated from a buffy coat (National Health Service Blood and Transplant, Bristol, UK) and early erythroblasts were expanded for one week in 10% conditioned medium (CM) from the bladder cancer cell line 5637, 10% non-heat inactivated FBS (SLI, Salisbury, UK) and 1μg/ml Cyclosporin A (Sandoz Pharmaceuticals, Surrey, UK) in α-modified MEM. Erythroid cells were washed twice before transfer into phase II medium comprising α-MEM with 1U/ml recombinant Epo (Ortho Biotech Janssen Cilag Ltd, Bucks, UK), 10ng/ml SCF (R&D Systems, Abingdon, UK), 0.3 mg/ml holo-transferrin (MP Biomedical, London, UK), 1μM dexamethasone (Faulding Pharmaceuticals plc, Warwickshire, UK), 3% non-heat inactivated FBS (SLI) and 1% deionized fraction V BSA. Erythroid precursors differentiate into mature erythroblasts and hemoglobinized normoblasts over 14 days. On day 6 of this second phase the cultures are enriched for erythroid progenitors using magnetic bead isolations (Miltenyi Biotec). Briefly CD3^+^ cells were depleted by a LD column and CD71^+^ cells selected from the excluded fraction using an LS column. Eluted cells (>90% viable) from the final step were incubated in freshly prepared Phase II medium with hemozoin or TNF-α for 24 hours before harvesting for RNA extraction. Lineage content of cells used for each experiment is shown in supporting information (S1 Tables).

### Isolation and preparation of malarial pigment

Mycoplasma-free *P*. *falciparum* (3D7) cultures were enriched for trophozoites and schizonts over 60% Percoll [60] and lysed in 8μg/ml digitonin for 10’ on ice. Centrifugation at 16400xg for 10’ at 4°C was followed by sterile sonication (Soni prep 150, MSE) in 2% SDS Tris pH8 for 2s at amplitude of 10 microns on ice. Hemozoin was obtained after 4–5 washes in 2% SDS Tris pH8, one wash in 1% Triton X-100 and finally three washes in 100-fold volumes of PBS, before storage under nitrogen at -80°C. Before use all pigment preparations were sonicated for 10–20s as described above.

### Quantification of pigment

The concentrations of hemozoin were determined following depolymerization in 20mM NaOH for 2 hours at room temperature [61]. The absorbance at 405nm was compared with known concentrations of α-hematin and the concentration determined as hematin equivalents/ml.

### RNA extraction, biotinylation of cRNA preparation and hybridization

Total RNA using 7.5 x10^5^ cells per treatment from one donor was extracted using Trizol (Invitrogen, Paisley, UK) or the RNEasy Mini kit (Qiagen) in accordance with the manufacturer’s instructions. An additional phenol-chloroform step was included for preparations isolated using Trizol. RNA integrity was evaluated using an Agilent Bioanalyzer 2100 (Agilent Technologies, Palo Alto, CA). Only RNA with RIN>9 was processed further for microarray. Any contribution of RNA from dead cells would have resulted in poor RNA integrity with a RIN considerably lower than 9. Biotinylated fragmented cRNA was synthesized from 100ng of pooled RNA using the 2-Cycle cDNA Synthesis and the 2-Cycle Target Labelling and Control Reagent kits (Affymetrix, Santa Clara, CA), and hybridized to GeneChip Human Genome U133_Plus_2.0 arrays (Affymetrix) following the manufacturer’s recommendations.

### Analysis of microarray data

Cell intensity calculation and scaling was performed using GeneChip Operating Software (GCOS) (Affymetrix) and data analysed by the Robust Multiarray Average (RMA) method of Irrizarry [25] using the Bioconductor suite packages in R (www.bioconductor.org). Probes with low signal intensity in all samples were filtered out and the resulting probes analysed for differential expression in Excel (Microsoft). Expression data for genes of interest were hierarchically clustered by gene and by experiment using Average linkage and Pearson correlation with Genesis (Graz University of Technology) [62]. GeneCodis was used for gene ontology mapping and obtaining functional output from differentially expressed genes. This stringent analysis provides gene ontologies which are significantly over-represented in the list of modulated genes, to detect programs of gene expression changes relevant to key biological processes. Corrected hypothetical p values are generated after the removal of redundant terms and inclusion of modules that clearly define different biological functions. Gene names were determined from Affymetrix HGU133_plus_2.0 probe identifiers (PIDs) using BioDB.net. Hierarchical clustering was performed by gene and by experiment using Pearson correlation in Genesis. The array data has been submitted to GEO with Accession number GSE65577.

### RT PCR

cDNA was synthesized from 100ng RNA using the High Capacity cDNA Reverse Transcription Kit (Life Technologies, Paisely, UK) following DNAse treatment where applicable. Real time PCR was performed using TaqMan Universal PCR Master Mix and validated gene expression assays (Life Technologies) (Table 1). TaqMan cycling conditions used were according to the manufacturer’s instructions. Expression data for each transcript was normalized to that for the reference gene Ribosomal Protein L13A (RPL13A) [63], which did not change expression during erythroid development [26] nor upon exposure to TNF-α or hemozoin (See gene lists in S1 Dataset). Fold changes compared with media controls were determined using the comparative Ct method.

**Table 1:** Taqman gene expression assays used for RT- PCR.

Gene Symbol	Context sequence for inventoried assay	Ref Seq	Assay ID	Amplicon length
CTSE	CTACATGAGCAGTAACCCAGAAGGT	NM_148964.1	Hs00157213_m1	57
CSFR2	TGACCCAGCATGTCCAGCCTCCTGA	NM_000395.2	Hs00166144_m1	73
DDIT3	AACCTGAGGAGAGAGTGTTCAAGAA	NM_004083.4	Hs01090850_m1	78
DDIT4	CGGAGGAAGACACGGCTTACCTGGA	NM_019058.2	Hs01111686_g1	68
ESCO2	TGAAGTGTGACAGCCTTTTACACTT	NM_001017420.2	Hs00411577_m1	136
IFI27	TGCCCCTGGCCAGGATTGCTACAGT	NM_001130080.1	Hs00271467_m1	63
MAP7	GAGAAAGAAGCGACTTGAGGAGATT	NM_003980.3	Hs01009609_m1	91
RPL13A	AGACTGGGAAGATGCACAACCAAGG	NM_012423.2	Hs01926559_g1	105

The inventoried target sequence and NCBI reference sequence (Ref Seq) for each amplicon is shown.

### Flow cytometry

Erythroid cell maturation was determined by measuring expression of the transferrin receptor (CD71) and glycophorin A (CD235a) with the appropriate mAbs (Beckman Coulter plc, High Wycombe, UK and DAKO Cytomation Ltd, Ely, UK). Monocytes and macrophages were identified with murine anti-CD14 (Serotec, Oxford, UK), using appropriate isotype controls. Cells were stained in 0.5% BSA, 0.05% sodium azide in PBS (staining buffer) for 30’ at 4°C, washed twice and fixed in 2% paraformaldehyde. Live cells were identified from FSC and SSC profiles of 7-AAD (Becton Dickinson, Oxford, UK) negative unfixed cells. The absolute number of erythroid cells was determined by multiplying the viable cell count by the proportion of CD71^+^ and/or CD235a^+^ cells acquired. Countbright fluorescent beads (Invitrogen, Paisley, UK) were also used to determine erythroid numbers in culture. Cells were washed and analyzed on the same day by flow cytometry on a FACSCalibur or LSR II machine (Becton Dickinson) at a flow rate of <1500 events/s. Events were analyzed with WinMDI (http://facs.scripps.edu/software.html) and Weasel software (http://www.wehi.edu.au/cytometry/WEASEL.html).

## Supporting Information

S1 Dataset Gene lists and Tables A-K. Lists of genes up-regulated and down-regulated by each treatment; tables showing the fold changes in expression determined by microarray and derivation of co-annotations with associated gene ontologies.
(XLSX)

S1 Fig Hierarchical clustering based on all genes regardless of treatment. Experimental data described in text was clustered with data previously obtained from primary erythroblasts sorted according to stage of development (26).
(TIF)

S2 Fig Induction of CHOP protein in erythroblasts and apoptosis induction by hemozoin extracted from schizonts or following release into tissue culture. Induction of CHOP protein in pro- and intermediate erythroblasts by (A) hemozoin and (B) HNE. Response to thapsigargin (Sigma T9033; induces Ca^2+^ efflux from mitochondria at 3μM) is shown as positive control. (C) The proportion of annexin positive 7AAD^+^ erythroblasts induced by hemozoin extracted as described in materials and methods (Hz_per) is comparable to hemozoin extracted following schizont release in culture (Hz_scr). *p<0.05 compared with medium controls using an un-paired t-test.
(TIF)

S1 Tables Cell lineage content of erythroblast cultures before and after magnetic bead enrichment. (PDF)
