# Correction: Distinct Mechanisms of Inadequate Erythropoiesis Induced by Tumor Necrosis Factor Alpha or Malarial Pigment

**DOI:** 10.1371/journal.pone.0127124

**Published:** 2015-04-22

**Authors:** Abigail A. Lamikanra, Alison T. Merryweather-Clarke, Alex J. Tipping, David J. Roberts

The following information is missing from the Funding section: The research leading to these results has received funding from the European Community's Seventh Framework Programme (FP7/2007-2013) under grant agreement N° 242095.

The complete, correct funding information is as follows: This article summarizes independent research funded by the European Community's Seventh Framework Programme (FP7/2007-2013) under grant agreement N° 242095 and by the National Institute for Health Research (NIHR) under its Programme Grants for Applied Research Programme (Grant Reference Number RP-PG-0310-1004). The views expressed are those of the author(s) and not necessarily those of the NHS, the NIHR or the Department of Health. The funders had no role in study design, data collection and analysis, decision to publish, or preparation of the manuscript.
