# Supplementary figures and images for: Distinct Mechanisms of Inadequate Erythropoiesis Induced by Tumor Necrosis Factor Alpha or Malarial Pigment
Source: PLoS One. 2015 Mar 17;10(3):e0119836. doi: 10.1371/journal.pone.0119836 (PMC4363658; doi:10.1371/journal.pone.0119836)

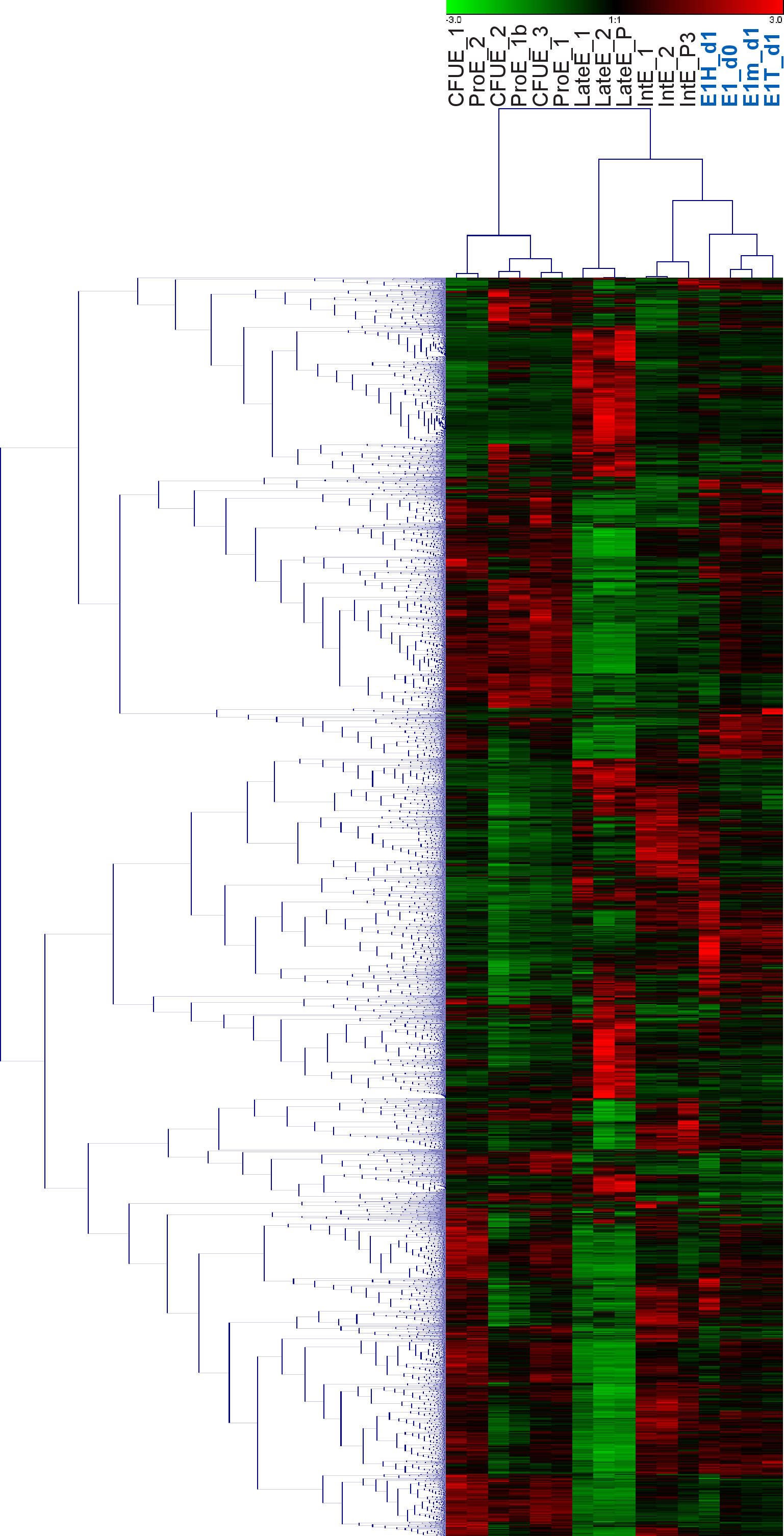

Supplement: S1 Fig — Experimental data described in text was clustered with data previously obtained from primary erythroblasts sorted according to stage of development (26). (TIF) [file pone.0119836.s002.tif]
