# Supplementary material for: Distinct Mechanisms of Inadequate Erythropoiesis Induced by Tumor Necrosis Factor Alpha or Malarial Pigment
Source: PLoS One. 2015 Mar 17;10(3):e0119836. doi: 10.1371/journal.pone.0119836 (PMC4363658; doi:10.1371/journal.pone.0119836)
Supplement: S1 Tables — (PDF) [file pone.0119836.s004.pdf]

Supporting Information: File 3

Table 1: Cell lineage content of erythroblast cultures **before** magnetic bead enrichment. Erythroid cells are defined as expressing CD71+CD235a+ or CD71+CD235a- and labelled with anti-bodies described in materials and methods. T and B lymphocytes were identified by flow cytometry using fluorochrome-conjugated antibodies specific to CD3 and CD19; macrophages were identified using antibodies specific to CD14.

Donor	Proportion of live cells (%)		
	Erythroid	Lymphoid	Macrophage
1	21	78	0.2
2	14	85	1.4
3	13	86	0.4
4	52	46	2.3

Table 2: Cell lineage content of cells used **following** magnetic bead enrichment for erythroid cells. The proportion of live events acquired is shown to demonstrate the biologically responsive component of the culture used for micro-array and qPCR (see materials and methods for details)

Donor	Use		Proportion of live cells (%)		
	Array	qPCR	Erythroid	Lymphoid	Macrophage
1	yes	yes	97	0.1	Not determined
2	yes	yes	98	0.2	Not determined
3	no	yes	94	0.7	Not determined
4	no	yes	95	2	1.5

Table 3: Viability of erythroblasts in untreated cultures 24 hours after magnetic bead isolation to demonstrate validity of culture conditions.

Donor	Use		Viable cells (%)
	Array	qPCR	Erythroid
1	yes	yes	97
2	no	yes	98
3	no	yes	99
4	no	yes	98
